# A Year in Review on Tuberculosis and Non-tuberculous Mycobacteria Disease: A 2025 Update for Clinicians and Scientists

**DOI:** 10.20411/pai.v10i2.791

**Published:** 2025-03-02

**Authors:** Christoph Lange, Graham Bothamley, Gunar Günther, Lorenzo Guglielmetti, Irina Kontsevaya, Liga Kuksa, Berit Lange, Natalie Lorent, Francesca Saluzzo, Martina Sester, Marc Tebruegge, Simone Tunesi, Conor Tweed, for the Tuberculosis Network European Trials group (TBnet)

**Affiliations:** 1 Infectious Diseases, Research Center Borstel, Leibniz Lung Center, Borstel, Germany; 2 Clinical Tuberculosis Unit, German Center for Infection Research (DZIF), Hamburg-Lübeck-Borstel-Riems, Germany; 3 Respiratory Medicine and International Health, University of Lübeck, Germany; 4 Baylor College of Medicine and Texas Children's Hospital, Global TB Program, Houston, Texas; 5 Homerton University Hospital, London, United Kingdom; 6 Queen Mary University of London, London, United Kingdom; 7 London School of Hygiene and Tropical Medicine, London, United Kingdom; 8 Department of Pulmonology, Allergology and Clinical Immunology, Inselspital Bern, Bern University Hospital, Bern, Switzerland; 9 Department of Clinical Sciences, School of Medicine, University of Namibia, Windhoek, Namibia; 10 Sorbonne Université, INSERM, U1135, Centre d'Immunologie et des Maladies Infectieuses, Cimi-Paris, Paris, France; 11 Assistance Publique Hôpitaux de Paris, Groupe Hospitalier Universitaire Sorbonne Université, Hôpital Pitié-Salpetrière, Centre National de Reference des Mycobactéries et de la Resistance des Mycobactéries aux Antituberculeux, Paris, France; 12 Department of Infectious Disease, Faculty of Medicine, Imperial College London, London, United Kingdom; 13 Tuberculosis and Lung Disease clinic, Riga East University hospital, Riga, Latvia; 14 Department of Epidemiology, Helmholtz Centre for Infection Research; 15 German Center for Infection Research, TI BBD; 16 Department of Respiratory Diseases, University Hospital Leuven, Leuven, Belgium; 17 Department of Chronic Diseases, Metabolism and Aging, Laboratory of Respiratory Diseases and Thoracic Surgery (BREATHE), KU Leuven, Leuven, Belgium; 18 IRCCS San Raffaele Scientific Institute, Milan, Italy; 19 Vita salute San Raffaele University, Milan, Italy; 20 Department of Transplant and Infection Immunology; 21 Center for Gender-specific Biology and Medicine (CGBM), Saarland University, Homburg, Germany; 22 Department of Child and Adolescent Medicine & Austrian National Reference Centre for Childhood Tuberculosis, Klinik Ottakring, Vienna Healthcare Group, Vienna, Austria; 23 Infectious Diseases Network, Vienna Healthcare Group, Vienna, Austria; 24 Department of Paediatrics, University of Melbourne, Parkville, Australia; 25 Infectious Diseases Unit, AOU SS Antonio e Biagio e C. Arrigo, Alessandria, Italy; 26 MRC Clinical Trials Unit, University College London, London, United Kingdom

**Keywords:** TB, NTM, pediatric TB, TBnet

## Abstract

**Background:**

In the field of tuberculosis and non-tuberculous mycobacterial (NTM) disease we are looking back on an exciting year 2024 with more than 10,000 publications listed in PubMed.

**Methods:**

Our aim, to review the scientific literature of the year 2024, is challenged by the enormous number of publications. Therefore, if your article is not included or your favorite field of mycobacteriology not covered, please forgive us. Our “Year in Review” is very much clinically oriented with lesser emphasis on basic science, microbiology, and biotechnology.

**Results:**

Members of the steering committee of the Tuberculosis Network European Trials group (TBnet; www.tbnet.eu) report on 139 publications in the fields of epidemiology, prevention, diagnosis, and treatment of tuberculosis and NTM diseases published in 2024 that we found particularly important. We report publications separately for tuberculosis in children and adults and for NTM disease and provide a brief overview of newer technologies in the diagnostic pipeline. Furthermore, we summarize priorities for tuberculosis and NTM disease research, development, and implementation, all of which represent the perspective of our combined clinical experience.

**Conclusions:**

This Year in Review provides a concise summary of the clinically relevant highlights of the published literature in tuberculosis and NTM diseases in 2024.

## INTRODUCTION

Tuberculosis is the leading infectious cause of death worldwide. Never before were more individuals reported by the World Health Organization (WHO) to be affected by tuberculosis than currently [1]. However, the burden of disease is not equally distributed [1]. In low tuberculosis incidence countries, infections with non-tuberculous mycobacteria (NTM) are on the rise, overtaking tuberculosis cases in terms of absolute numbers in some regions where tuberculosis is almost eliminated, like Denmark, Scotland, the United States, and Canada[2–4].

Today, innovations in the area of anti-tuberculosis vaccines, novel diagnostics, and new medicines in phases I to III of clinical evaluation are in progress. We have reviewed the literature on mycobacterial infections published in 2024 and present the latest developments in epidemiology, prevention, diagnostics, and treatment of tuberculosis and NTM diseases in adults and children.

## EPIDEMIOLOGY

**Figure 1.**
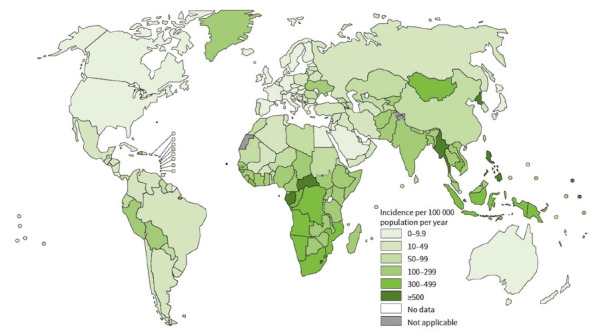
Estimated incidence of tuberculosis in 2023 (courtesy of the WHO [1])

According to the World Health Organization (WHO) global tuberculosis report 2024, there were 10.8 million (95% uncertainty interval [UI]: 10.1–11.7) individuals falling ill with tuberculosis in the year 2023, which represents a continued increase from 10.1 million in 2021 [1] (Figure 1). This corresponds to a global incidence of 134 per 100,000 population (95% UI: 125–145), one of the highest incidences ever reported globally, not explained by the increase in population alone. Although the case detection gap is closing, only 8.2 million people were reported as newly diagnosed with tuberculosis in 2023, leaving 2.6 million individuals as estimated cases [1].

Reductions in tuberculosis deaths since 2022 and slowing increase in the tuberculosis incidence are attributed to substantial post-COVID recovery in tuberculosis diagnosis and treatment [1].

The incidence of tuberculosis is variably distributed with an estimate of more than 500 cases per 100,000 in the Central African Republic, the Democratic People's Republic of Korea, Gabon, Lesotho, Myanmar, and the Philippines, compared with 3.1 per 100,000 population (95% UI: 2.6–3.6) in the United States and 8.6 per 100,000 population (95% UI: 8.2–9.0) in the European Union/European Economic Area, where tuberculosis is trending towards elimination. The 5 countries with the highest burden of disease include India (26%), Indonesia (10%), China (6.8%), the Philippines (6.8%), and Pakistan (6.3%), which account for more than half (56%) of all cases globally.

An estimated 12% (1.3 million; 95% UI: 1.2–1.3 million) of those who developed tuberculosis in 2023 were children and young adolescents (aged 0–14 years), groups that are especially vulnerable to severe disease and in whom microbiological confirmation is often challenging. Overall, an estimated 1.25 million individuals died from tuberculosis in 2023 (95% UI: 1.13–1.37 million) [1].

Antimicrobial resistance in *Mycobacterium tuberculosis* is of increasing concern [5–8]. According to data from the Antimicrobial Resistance Collaboration, rifampicin-resistant tuberculosis ranks seventh among all deaths caused by drug-resistant microorganisms. It is also the only drug-resistant organism on the list with an annualized rate of change exceeding 5% between 1990 and 2021 [9]. In Summer 2024, the WHO published a list of bacterial pathogens of public health importance to guide research and strategies to prevent and control antimicrobial resistance. Rifampicin-resistant tuberculosis is in the top category of 4 globally critical pathogens [10]. The estimated number of people affected by multidrug-resistant or rifampicin-resistant tuberculosis (MDR/RR-TB) in 2023 was 400,000 (95% UI: 360.000–440.000), of whom only 175,923 (44%) received treatment for drug-resistant tuberculosis (DR-TB). Tuberculosis treatment coverage in 2022 was estimated to be at 75% (95% UI: 70–81%), and 88% of patients treated for tuberculosis achieved successful treatment outcomes. In contrast, treatment coverage for patients with MDR/RR-TB was only 44% with a global treatment success rate of only 68% [1]. Successful treatment outcome was only achieved in 25% of 52 patients affected by extensively drug-resistant tuberculosis in Georgia, Kazakhstan, Kyrgyzstan, Moldova, and Ukraine during 2017–2022 [11].

Efforts for tuberculosis prevention are still inadequate. Asymptomatic tuberculosis is becoming recognized as an important part of the tuberculosis disease spectrum that needs to be adequately addressed by any prevention strategy [12–14]. Although there was an increase in the proportion of household contacts receiving tuberculosis preventive therapies in 2023 compared to previous years, the estimated global coverage of tuberculosis preventive treatment among household contacts reached only 21% [1].

While a large proportion of dynamic modelling studies of tuberculosis are rightly focused on pharmaceutical interventions, social determinants of tuberculosis burden have been increasingly on the agenda. Historical data from Denmark covering the past 150 years show that most of the decrease in tuberculosis incidence was achieved by improving social determinants, not by anti-tuberculosis medicines [15]. Poverty significantly increases the risk of developing tuberculosis, which in turn can further worsen economic hardship for affected families. [16].

The war in Ukraine has led to a 4-fold increase of DR-TB in the European Union/European Economic area (EU/EEA) with most countries being ill-prepared for the challenges of tuberculosis screening in migrants [17]. According to a recent study by TBnet, migrants to the EU/EEA and the UK are at increased risk of extrapulmonary tuberculosis, DR-TB, and tuberculosis / HIV co-infection, and they experience worse treatment outcomes than non-migrant host populations [18].

Not only war and migration, but also incarceration may have a large effect on the epidemiology of tuberculosis. In Latin America, 30% of the tuberculosis burden has been attributed to increasing incarceration rates since 1990 [19].

The first edition of the Global Tuberculosis Dictionary intends to clarify and streamline terminology related to tuberculosis epidemiology and other related topics and by doing so introduces consistency and effective communication in the field. For example, the term active tuberculosis is frequently used to denote a person with disease caused by *M. tuberculosis*, to contrast with the traditional term, latent tuberculosis infection. However, we now know that so-called latent tuberculosis is not truly inactive tuberculosis, or even latent as understood in other fields [20].

## HIV, VIRAL HEPATITIS, DIABETES, AND OTHER COMORBIDITIES

Communicable and non-communicable diseases have an impact on tuberculosis management and contribute to an increase in unfavourable outcomes of tuberculosis treatment. In 2024, the WHO released a consolidated guideline about tuberculosis and HIV co-infection and tuberculosis and mental health [21].

HIV prevalence among patients infected with *M. tuberculosis* remains substantial, although it is constantly decreasing. In 2023, only 6.1% of new tuberculosis cases worldwide were people living with HIV (PLHIV). The global distribution of HIV coinfections remains unbalanced, with almost 50% of coinfected patients residing in the WHO African Region. An estimated 161,000 PLHIV died of tuberculosis in 2023. For the second consecutive year, the mortality trend appears to be decreasing after the COVID-19 pandemic period [1]. There is convincing evidence that ongoing HIV replication, including suboptimal HIV control with antiretroviral therapy, is an important risk factor for tuberculosis development in PLHIV, independent of CD4 cell count [22].

Considering the global epidemiology, much effort in early diagnosis has been put into low-resource/high-prevalence countries. Early diagnosis of tuberculosis and HIV infections and implementation of tuberculosis infection treatment remain major topics. The SEARCH study showed that in rural areas in Uganda universal HIV testing was a valuable strategy for reducing tuberculosis transmission [23]. Use of lateral-flow lipoarabinomannan (LAM) tests on urine could have an important effect in averting tuberculosis deaths amongst PLHIV with advanced disease by earlier identification and treatment [24]. The EXULTANT trial now showed that a urine LAM test improved early tuberculosis diagnosis in hospitalized asymptomatic patients with HIV infection [25]. More evidence about the effectiveness of same-day treatment policies is expected to emerge in the coming years [26]. An effect on improvement of morbidity and mortality is expected, but still needs to be demonstrated.

Co-infections caused by HBV and HCV remain a common cause of comorbidity and lead to an increased risk of hepatotoxicity in patients with *M. tuberculosis* infection. Treatment monitoring is essential, eg, to identify hepatotoxicity by elevated serum transaminases and/or serum bilirubin [27]. Moreover, data from Californian cohorts show that even in high-income/low-prevalence countries, detection of concomitant *M. tuberculosis* infection (TBI) and chronic HBV infection is a relative common finding [28], and in the same setting the prevalence of coinfection in foreign-born individuals is higher [29].

Mental health in tuberculosis patients is an important field of research and action. In some specific populations, such as prisoners [30] and migrants [31], taking care of mental health conditions is a major part of tuberculosis management. Tuberculosis-related stigma seems to be more common in people affected by DR-TB [32], leading to anxiety and depression that can adversely affect treatment adherence and outcomes [33].

Diabetes increases the risk of tuberculosis in the short and long term [34]. Even impaired fasting glucose seems to be a risk factor for active tuberculosis, indicating that tuberculosis should be screened for in diabetic patients — at least in high-tuberculosis-prevalence settings [35]. Recently commenced studies may help to better elucidate the link between hyperglycemia and tuberculosis, [36] in order to improve tuberculosis prevention and management through better control of blood sugar levels.

## IMMUNOLOGY AND PREVENTION

Prevention of tuberculosis relies on early identification of individuals at risk. In an effort to standardize terminology and definitions regarding asymptomatic forms of disease, the WHO recommended using the term asymptomatic tuberculosis [1], which is also referred to as subclinical tuberculosis in recent publications. Given the absence of symptoms, estimating the prevalence of asymptomatic tuberculosis stages clearly depends on efforts towards their identification.

An international Delphi exercise has developed a minimum consensus to define the spectrum of infection with *M. tuberculosis* based on infectiousness, symptoms, and macroscopic pathologies [12]. *Mycobacterium tuberculosis* infection was defined as a non-infectious state without symptoms or pathology. Subclinical (asymptomatic) tuberculosis was defined as states with macroscopic pathologies in the absence of symptoms. Finally, clinical tuberculosis was defined as a state with both symptoms and macroscopic pathologies. Both subclinical (asymptomatic) and clinical tuberculosis can exist in 2 subcategories based on likely infectiousness. This path towards refined pragmatic definitions will allow better standardization of target groups for interventions. The results of a recent meta-analysis emphasize the substantial burden of subclinical (asymptomatic) tuberculosis in high tuberculosis prevalence countries, especially its contribution to transmission [14]. Although technically challenging, [^18^F]-fluorodeoxyglucose positron emission tomography/computed tomography ([^18^F]FDG-PET/CT) scans in combination with targeted detection of *M. tuberculosis* at sites of increased metabolic activity, and low-level mycobacterial DNA in the blood have been shown to identify household contacts with early stages of subclinical (asymptomatic) tuberculosis [37]. Moreover, changes in *M. tuberculosis*-specific antibody profiles have been identified in progressors up to 2 years before developing active tuberculosis [38]. Interestingly, a better correlation of the profile with progression was observed in adolescents than among adults and in males compared with females.

The effectiveness of preventive treatment in contacts of patients with active tuberculosis has been analyzed based on an individual participant data meta-analysis [39]. A total of 439,644 contacts in both high- and low-tuberculosis burden countries across all age groups were included. Overall effectiveness was 49% when comparing covariate-adjusted risk of incident tuberculosis between groups to describe the protective effectiveness of preventive treatment. Of note, effectiveness was higher among individuals with a positive interferon-gamma release assay (IGRA) or tuberculin skin test (TST), and among contacts in high-burden countries. Preventive treatment was ineffective for contacts who tested negative by IGRA or TST, except for children under 5 years of age. Among contacts from high-burden countries, the effectiveness of prevention remained high regardless of IGRA or TST test results. Overall, these results may further support a treat all approach for contacts below the age of 5 years and from high-burden countries. In low-burden countries, the number needed to treat to prevent a contact person from developing tuberculosis may be substantially decreased, if targeted preventive treatment is based on a positive TST or IGRA. A treat all approach is easier to implement with availability of shorter regimens for preventive treatment. In this regard, a randomized trial has shown similar safety but slightly lower completion rates of a 2-month rifampicin regimen of 20 mg/kg daily dose than the standard 4 months regimen of 10 mg/kg daily dose [40]. In contrast, severe adverse events and completion rates of a 2-month regimen of 30 mg/kg rifampicin were unacceptably unfavorable [40]. Pending confirmation of similar efficacy, with data being expected soon, the 2-month 20 mg/kg daily regimen may represent an attractive alternative to longer treatment regimens for tuberculosis prevention.

This year has also witnessed progress in our knowledge on prevention of tuberculosis among contacts of patients with MDR/RR tuberculosis. Two double blind placebo controlled studies, one mainly among adults in Vietnam [41] and one among children in South Africa [42], have shown that a 6-month course of once-daily levofloxacin was associated with lower numbers of tuberculosis cases as compared to placebo. Although the differences in each individual study were not significant, a meta-analysis of the 2 studies provided evidence for a 59% relative reduction in the cumulative incidence of tuberculosis over 1 year [43], which is in line with a 66% risk reduction by tuberculosis preventive therapy (TPT) among contacts with MDR/RR-tuberculosis based on another meta-analysis of 11 cohort studies that included different TPT regimens [44]. In this meta-analysis subgroup analyses showed that the resistance profile-guided regimen of TPT had a significant protective effect against the development of TB disease, whereas the uniform treatment regimen did not exhibit a statistically significant effect [44]. Despite absence of grade 3 adverse events, levofloxacin TPT has been associated with more grade 1 or 2 events, which may affect its adherence in clinical practice [45].

Further prevention strategies, including a comprehensive overview of current vaccines in development have recently been reviewed [46]. Vaccines currently in clinical development are summarized in Table 1. While a prevention of disease phase III trial of the most promising adjuvanted M72/AS01e vaccine is currently being conducted in individuals with a positive IGRA (NCT06062238), a recent study has confirmed conflicting evidence suggesting that Bacille Calmette-Guérin (BCG) revaccination plays a limited role in preventing infection, as determined by IGRA conversion, among healthcare workers at risk of exposure [47]. Moreover, the safety and immunogenicity of a ChAdOx1 85A priming followed by MVA85A boosting vaccine combination was evaluated in a randomized trial against BCG revaccination in Ugandan adolescents who had received BCG vaccination at birth [48]. The prime-boost regimen was well tolerated and induced superior cellular and humoral immune responses against antigen 85A and similar responses towards PPD, although it remains uncertain how this translates into protective efficacy. In this regard, a meta-analysis of individual participant data found protection against IGRA conversion by primary BCG vaccination among household contacts that was shown to be consistent with protection from developing tuberculosis [49]. However, correlates of protection from tuberculosis are still ill-defined. If confirmed, these data may hold promise for development of IGRA-based endpoints accelerating selection of novel vaccine candidates. As a further innovative approach to accelerate vaccine development, a phase I trial has studied the safety of an aerosol BCG human infection model, which was well-tolerated and may hold promise as a model for assessing vaccine efficacy and correlates of protection [50].

**Table 1.** Major Tuberculosis Vaccine Candidates in Clinical Development (updated from [46] with permission)

Name	Composition	Most advanced clinical stage	Representative clinical trial number
**TB Protein: adjuvant formulations**			
H56:IC31	Fusion protein of 2 antigens: IC31 as adjuvant^1)^	Phase IIb ongoing	NCT03512249
ID93:GLA-SE	Fusion protein of 4 antigens: GLA-SE as adjuvant^2)^	Phase IIb ongoing	NCT03806686
M72:AS01_E_	Fusion protein of 2 antigens: AS01_E_ as adjuvant^3)^	Phase IIb completed	NCT01755598
M72:AS01_E_-4	Fusion protein of 2 antigens: AS01_E_ as adjuvant^3)^	Phase III ongoing	NCT06062238
AEC:BC02	Combination of 3 protein antigens: BC02 as adjuvant^4)^	Phase IIa ongoing	NCT05284812
GamTBvac	Combination of 3 protein antigens: CpG as adjuvant^5)^	Phase III ongoing	NCT04975737
**Mtb-antigen encoding mRNA vaccines**			
BNT164a1 / BNT164b1	mRNA expressing multiple Mtb antigens in lipid nanoparticles	Phase I ongoing	NCT05547464 NCT05537038
**TB antigen expressing viral vectors**			
ChadOx1.85A/MVA85A	ChadOx1 as carrier for prime, MVA as carrier for boost, both expressing same antigen^6)^	Phase IIa completed	NCT0368160
TB/FLU-04L	Non-replicating influenza virus expressing 2 antigens^7)^	Phase I completed	NCT02501421
**Inactivated whole cell vaccines**			
Immuvac	Killed *M. indicus pranii*	Phase III ongoing	CTRI/2019/01/017026
RUTI	Killed detoxified *M. tuberculosis*	Phase IIb ongoing	NCT04919239
DAR-901	Killed *M. obuense*	Phase IIb completed	NCT02712424
**Viable attenuated whole cell vaccines**			
MTBVAC	Genetically attenuated *M. tuberculosis*^8)^	Phase III ongoing	NCT04975178
VPM1002	Genetically improved BCG^9)^	Several phase III ongoing	NCT04351685

1 IC31 adjuvant: cationic peptide with a TLR-9 agonist.

2 GLA-SE: oil-in-water emulsion with TLR-4 agonist.

3 AS01_E_: liposome with TLR-4 agonist.

4 BC02: CpG adjuvant in aluminum hydroxide.

5 CpG adjuvant.

6 Chimpanzee adenovirus (ChadOx1) as prime and modified vaccinia Ankara (MVA) as boost both expressing same antigen.

7 Non-replicating influenza virus expressing 2 antigens.

8 2 independent gene deletions (phoP and fadD26) in Mtb.

9 Exchange of urease C by listeriolysin gene in BCG.

## DIAGNOSTICS

New WHO guidelines have finally endorsed the use of targeted next-generation sequencing (tNGS) for the determination of drug resistance in people with bacteriologically confirmed pulmonary tuberculosis in 2024 [51]. These new recommendations are expected to improve the tuberculosis diagnostics pathway, especially in DR-TB high-burden countries where tNGS has already been considered a possible addition to current diagnostic algorithms [52]. Phenotypic methods are becoming more standardized, even for new compounds entering clinical trials, thanks to the work of several groups, including EUCAST [53]. Nonetheless, the interpretation of resistance for new compounds can be difficult to establish [54].

The evolution of technologies and the upsurge of artificial intelligence (AI)-based tools are leading to a shift in tuberculosis diagnostics. AI-powered computer-aided diagnostics, including chest x-ray, are now a reality in several countries [55], and the potential of AI cough tools — AI-powered monitoring of cough counts as a prediction of tuberculosis and/or treatment progression and AI-powered classification of cough sounds for TB screening — to supplement tuberculosis diagnostic pathways is widely recognized [56].

Non-sputum-based tuberculosis diagnostic tools play an increasing role in individuals with paucibacillary disease, such as children or PLHIV, in whom obtaining a sputum sample is challenging [57]. Tongue swab collection is one of the approaches that has seen progress, and several studies have demonstrated its increasing sensitivity and high specificity for the direct detection of *M. tuberculosis* using commercially available and in-house PCR assays [58, 59]. Strategies to maximize sensitivity of the method and automate sample processing are being investigated [60].

The analysis of exhaled breath [61] is a non-invasive approach that may be particularly valuable in childhood TB. Various devices, such as e-nose or exhaled breath condensate collection devices, can detect exhaled volatile or non-volatile organic compounds [62, 63]. Also, *M. tuberculosis* DNA can be detected directly in the exhaled breath utilizing a face mask [64]. However, the performance of exhaled breath assays remains suboptimal and currently unsuitable in children, as discussed under “Tuberculosis in Children” [65].

Urine represents another promising non-invasive biomaterial for tuberculosis diagnostics, and several studies have evaluated the feasibility of detection of *M. tuberculosis* DNA in children and adults, including PLHIV [66, 67]. A more widely used alternative, especially in PLHIV, is the detection of LAM in urine, although performance remains suboptimal [68, 69].

As further discussed under “Tuberculosis in Children”, childhood tuberculosis is increasingly leveraging non-sputum-based diagnostic tools [57, 70, 71], as exemplified by improving performance of tools detecting *M. tuberculosis* in stool [72, 73].

Nevertheless, sputum remains important, especially in treatment monitoring. A new chemiluminescent enzyme immunoassay able to rapidly quantify LAM in sputum has been reported to have potential value for treatment monitoring [74]. The molecular bacterial load assay is gaining importance for the same purpose [75, 76], and a new commercial kit is soon to be on the market.

The diagnostic accuracy of novel interferon-γ release assays — alone [77] and in combination with other tools [78] — for the diagnosis of LTBI is currently under evaluation.

Tools based on the assessment of the expression of specific genes and their combinations have been developed and evaluated for the diagnosis of tuberculosis infection and active tuberculosis [79–82], predicting progression from asymptomatic to clinical tuberculosis [83, 84], and for treatment monitoring [85]. Moreover, the detection of cell free DNA and RNA from peripheral blood appears to be a promising tool for the early detection of pulmonary and extrapulmonary tuberculosis, despite being in the early phases of development [86, 87]. Unfortunately, research for new tests of progression from tuberculosis infection to symptomatic tuberculosis is still stagnating. Table 2 provides an overview of the tuberculosis diagnostics pipeline.

**Table 2.** Tuberculosis Diagnostics Pipeline Overview (modified from [88])

Type	Proof of concept / early evaluation	Analytical & clinical verification	Clinical validation	Commercially available / WHO-recommended
Screening tests		Cough sound apps		Chest x-ray + computer-aided detection
Diagnostic tests	Point-of-care rapid molecular tests for TB	Near-point-of-care rapid molecular tests for TB & DR-TB Point-of-care urine LAM tests for TB		Targeted next-generation sequencing
Treatment monitoring tests	LAM tests on sputum	Quantitative bacterial load tests Host-response tests		
TB infection tests			Point-of-care IGRA	Next-generation skin tests

DR-TB, drug-resistant tuberculosis; IGRA, interferon-gamma release assay; LAM lipoarabinomannan; TB, tuberculosis.

## TREATMENT OF DRUG-SUSCEPTIBLE AND DRUG-RESISTANT TUBERCULOSIS

The year 2024 has been a landmark period for research on the treatment of tuberculosis, with several published studies that advance both clinical understanding and treatment strategies.

### Drug-susceptible Tuberculosis

Results of a clinical trial evaluating the 4-month BPaMZ regimen (bedaquiline, pretomanid, moxifloxacin, and pyrazinamide) indicated significant treatment-shortening potential, with sputum culture conversion achieved in 84% of participants by week 8, compared to 47% with the 6-month HRZE (isoniazid, rifampicin, pyrazinamide and ethambutol) control [89]. However, BPaMZ did not reach non-inferiority to the control resulting from high withdrawal rates due to hepatic adverse events. These results confirm multiple previous findings of increased liver toxicity associated with the concomitant use of pretomanid and pyrazinamide [90–92].

A secondary analysis of the TBTC study 31/ACTG A5349, which showed non-inferiority of a 4-month rifapentine/moxifloxacin-based regimen to 6 months of HRZE, identified 3 major predictors of tuberculosis-related unfavorable outcomes: low rifapentine exposure (in the rifapentine-moxifloxacin arm), low Xpert MTB/RIF cycle threshold, and greater extent of disease on baseline chest radiography. These factors may allow stratifying TB phenotypes into hard- and easy-to-treat variants, opening relevant perspectives towards personalized medicine [93].

A phase IIa trial evaluating ganfeborole (GSK3036656), a novel leucyl-tRNA synthetase inhibitor, demonstrated significant early bactericidal activity against drug-susceptible *M. tuberculosis*, with the 30 mg dose showing the highest reduction in colony-forming units [94]. The drug was well-tolerated with no serious adverse events and exhibited dose-proportional pharmacokinetics. A post-hoc exploratory computational analysis of [^18^F]FDG-PET/CT findings demonstrated measurable treatment responses across multiple lesion types in patients receiving ganfeborole (30 mg) on day 14. Additionally, whole-blood transcriptional analysis on day 14 revealed a strong association between treatment response and neutrophil-dominated transcriptional modules [94]. These findings highlight ganfeborole's potential as a future component of tuberculosis treatment regimens, particularly for drug-susceptible tuberculosis, while also suggesting potential applicability in DR-TB with further research.

Updated WHO Target Regimen Profiles (TRPs) set ambitious new goals for drug-susceptible tuberculosis (DS-TB) regimens, emphasizing shorter, more effective treatments with minimal adverse effects [95]. These targets include achieving treatment durations of 4 months or less, ensuring efficacy rates above 90% for drug-susceptible cases, minimizing adverse events such as hepatotoxicity, and simplifying dosing schedules to improve patient adherence and programmatic feasibility. Revised TRPs include specific targets for drug efficacy, safety, and administration simplicity, guiding developers toward patient-centered innovations.

Modeling studies evaluated the financial feasibility of implementation of novel, shorter DS-TB regimens in India, South Africa, and the Philippines [96]. These models revealed that shorter-duration regimens could be cost-neutral or even cost-saving due to reduced patient monitoring needs, lower relapse rates, and decreased healthcare system burden. Cost-effective drug pricing and streamlined care delivery emerged as critical factors for successful implementation.

Innovative adherence monitoring tools, including 99DOTS (https://www.99dots.org/About.html), a digital adherence technology using medication sleeves with which patients interact during treatment, and video-observed therapy (VOT), which leverages video technology to monitor medication intake, were evaluated for cost-effectiveness in drug-sensitive tuberculosis management [97]. These technologies enhance patient adherence by enabling remote monitoring, reducing the need for frequent clinic visits, and providing real-time data to healthcare providers. This support helps ensure treatment completion and reduces the risk of relapse or development of drug resistance. While 99DOTS showed comparable costs to standard DOT in some settings, VOT's higher implementation costs were offset by reduced patient monitoring expenses in scale-up scenarios.

### Drug-resistant Tuberculosis

Emerging clinical research evidence contributed to further shape the rapidly evolving field of DRTB treatment [98, 99]. With 19 novel anti-tuberculosis drugs in clinical phases I to III of development at the end of 2024, the tuberculosis “drug pipeline” has never been more promising before [100] and includes entire new drug classes [94].

The final results of the TB-PRACTECAL trial confirmed the safety and efficacy of the 6-month combination of bedaquiline, pretomanid, linezolid, and moxifloxacin (BPaLM), which became the standard of care for the treatment of rifampicin-resistant tuberculosis (RR-TB) in 2022 [101]. A secondary analysis of cardiac safety results from this trial showed that overall QTcF interval prolongation was slightly higher in the treatment arm including clofazimine (BPaLC) compared to BPaLM, although the difference was not clinically meaningful [102]. While the optimal dosing regimen of linezolid in RR-TB regimens is still unclear, a recent randomized trial performed in India suggested that, when using a 6–9-month BPaL regimen, a structured linezolid dose reduction to 300 mg/day after 2 or 3 months of treatment may have similar efficacy as the standard 600 mg/day dose, with a trend towards lower rates of peripheral neuropathy [103]. Still 1.5 years after the recommendation by the WHO in favor of the BPaLM regimen only half the countries in the WHO Europe Region had all medicines of the regimen available and drug susceptibility testing for all medicines of the regimen implemented [104].

**Figure 2.**
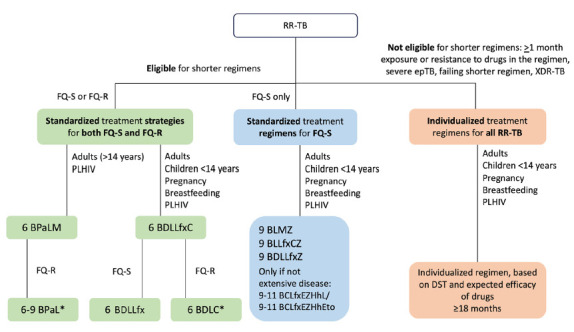
Summary of treatment recommendations for multidrug-resistant/rifampicin-resistant tuberculosis (MDR/RR-TB), pre-extensively resistant tuberculosis (pre-XDR-TB = RR-TB with additional resistance to any fluoroquinolone) and extensively resistant tuberculosis (XDR-TB = pre-XDR-TB with additional resistance to bedaquiline and/or linezolid) by the World Health Organization at the end of 2024. * = these regimens for fluroquinolone-resistant, rifampicin-resistant tuberculosis are recommended based on very low certainty of evidence, coming from studies which were either uncontrolled or included both fluoroquinolone-susceptible and fluoroquinolone-resistant tuberculosis cases, with the latter representing only a small subgroup. FQ-S, fluoroquinolone-susceptible; FQ-R, fluoroquinolone-resistant; epTB, extrapulmonary tuberculosis; PLHIV, people living with HIV; 6, 9, 11, treatment duration in months; B, bedaquiline; Pa, pretomanid; L, linezolid; M, moxifloxacin; D, delamanid; Lfx, levofloxacin; C, clofazimine; Z, pyrazinamide; E, ethambutol; Hh, high-dose isoniazid; Eto, ethionamide; DST, drug susceptibility testing.

Compelling evidence emerged on the efficacy of new 9-month, all-oral treatment regimens for fluoroquinolone-susceptible RR-TB. A large, prospective interventional cohort in the WHO Europe region reported excellent outcomes of 3 regimens including clofazimine, levofloxacin, and linezolid, plus bedaquiline and either cycloserine or delamanid for adults, and delamanid alone for children [105]. These promising results were supported by operational research studies evaluating similar 9-month all-oral regimens in adults with fluoroquinolone-susceptible RR-TB in Kazakhstan [106] and in children with both fluoroquinolone-susceptible and fluoroquinolone-resistant RR-TB in Afghanistan [107]. New treatment options, also including results from Médecins sans Frontière's (MSF) endTB trial [108], were endorsed by the WHO as part of a “Rapid Communication” announcing future changes to the guidelines on the treatment of DR-TB [109]. Figure 2 provides an overview of current WHO-endorsed treatment recommendations for different levels of RR-TB.

Two retrospective analyses of large cohorts of patients affected by RR-TB shed further light on treatment with long, individualized regimens. In a first study, sputum culture reversion to positivity after an initial conversion was found to be associated with cavitary lung disease, low body mass index, HCV infection, previous treatment with second-line drugs, and longer time to initial culture conversion [110]. A second study identified the association of sputum smear positivity and lung cavities at baseline as a key predictor of mortality and failure/recurrence among patients treated for RR-TB [111], supporting previous analyses of data from drug-susceptible clinical trials [112].

In the field of isoniazid-resistant tuberculosis, a phase II early bactericidal activity trial found that high-dose isoniazid (15–20 mg/kg) did not have significant bactericidal activity in the presence of a *katG* mutation [113], which is highly prevalent among multidrug-resistant *M. tuberculosis* strains. This complements previous findings showing the activity of high-dose isoniazid in *inhA*-mutated strains [114].

While many new treatment regimens are currently available to treat most RR-TB cases, patients suffering from extensively drug-resistant tuberculosis (XDR-TB) or tuberculosis with expanded resistance (defined as resistance to any new or repurposed second-line tuberculosis drug) are often left with limited treatment options and require longer, individualized regimens. The BETTER Field Guide, produced by a large and diverse group of clinicians and RR-TB experts, represents a first attempt to provide much-needed guidance for the treatment of these forms of tuberculosis, while awaiting emerging evidence and better treatment alternatives [115].

## TUBERCULOSIS IN CHILDREN

A study, using data from the European Centre for Disease Prevention and Control (ECDC), which focused specifically on migrant children and young people (CYP) and included more than 70,000 individuals <17 years of age with tuberculosis, found that across the EU (+ Iceland, Liechtenstein, Norway, and Switzerland) region more than 75% were native-born CYP, and less than 25% were migrant CYP [116]. Country-based analyses revealed that, while in Eastern European countries native-born CYP with tuberculosis far outnumbered those who were migrant CYP, the reverse was true in most Western and Northern European countries. Moreover, migrant CYP had an almost 2-fold higher risk of experiencing unsuccessful treatment outcomes, highlighting that urgent action is needed to rectify this disparity.

A unique birth cohort study from South Africa has provided further, strong evidence that the incidence of tuberculosis infection and disease remains substantially underestimated in high tuberculosis prevalence settings [117]. The authors found that the cumulative hazard of tuberculosis infection (based on TST conversion) was 36% at age 8 years, while the cumulative hazard of tuberculosis was 10% at age 10 years. The study also provided robust evidence that preventive treatment in children who experience TST conversion substantially reduces the risk of developing tuberculosis (adjusted hazard ratio: 0.23).

A multi-center study on tuberculosis in immunocompromised children — including children with primary immunodeficiencies, HIV infection, and malnutrition — conducted by the Paediatric Tuberculosis Network European Trials group (ptbnet), provided new insights into this particular patient group [118]. The authors found that microbiological tests for tuberculosis (ie, mycobacterial culture and molecular tests) perform as well in immunocompromised as in immunocompetent children, or even have higher yields, while the sensitivity of immune-based tuberculosis tests (ie, TST and QuantiFERON-TB Gold assay) was reduced significantly in the former group. Multivariate analyses revealed that presence of a primary immunodeficiency, malnutrition and a negative QuantiFERON-TB Gold assay result at presentation were associated with poor outcome, defined as death or chronic sequelae due to tuberculosis.

Achieving microbiological confirmation of tuberculosis in children remains substantially more difficult than in adults, in part owing to the difficulties in obtaining respiratory samples from young children. A recent study in adults showed that sampling via a purpose-built face mask can be effective and potentially determine the infectiousness of individual patients [119]. However, an exploratory study in children that also used face mask sampling reported disappointing results, leading the authors to hypothesize that this could be due to the typically paucibacillary nature of pediatric tuberculosis, the short sampling time employed in the study, or the suboptimal design of the face mask [65].

Several studies evaluated stool as a potential alternative to respiratory samples in children. One study, which included more than 500 children with presumed tuberculosis disease, evaluated stool and urine samples analysed with the Xpert Ultra assay and found that both sample types only achieved suboptimal sensitivity (stool validated against pulmonary samples: 69.4%; urine: 13.9%) [72]. Notably, if both confirmed and unconfirmed tuberculosis cases were included, the sensitivity of stool PCR was as low as 14.1% (42/298 cases). Another study including both adults and adolescents that evaluated the Xpert assay and a quantitative PCR performed on stool samples made similar observations [73]. Using a composite microbiological reference standard, the authors estimated the sensitivity of the assays in adolescents to be 69.6% and 73.9%, respectively. However, when clinical tuberculosis diagnosis was used as the reference standard, the estimated sensitivities of the assays were as low as 29.2% and 30.8%, respectively. Consequently, further optimization of stool processing and/or improvements in the design of molecular assays are required before stool samples could potentially replace respiratory samples in the diagnostic work-up for tuberculosis in children and adolescents.

One large study evaluated Cepheid's *Mycobacterium tuberculosis* Host Response prototype cartridge, which is based on the expression of 3 genes, but the reported sensitivity was too low (sensitivity against microbiological confirmation: 41.6%; CI, 34.7–48.7) for the test to be used in clinical practice [120]. However, modification of the assay, for example by inclusion of additional genes, may improve the performance of the assay to a level that is clinically useful.

The results of both the TB-CHAMP and the VQUIN MDR trials, both investigating whether chemoprophylaxis with levofloxacin can prevent active tuberculosis in MDR-TB contacts, were finally published in December this year [41, 42]. Although neither study individually showed a statistically significant reduction in active tuberculosis with preventive treatment compared to placebo, a pre-specified meta-analysis of both trials found that levofloxacin treatment reduced the cumulative incidence of tuberculosis by nearly 60% (to a relative difference in cumulative incidence of 0.41; 95% CI, 0.18–0.92; p=0.03)[43].

Several pediatric studies focused on post-tuberculosis lung disease — a subject that had received considerable attention in adult medicine over recent years — were published over the last year [121–123]. Overall, those studies showed that forced expiratory volume and forced vital capacity in children and adolescents were commonly reduced at treatment completion and up to one year thereafter. Further studies are required to determine the impact of those changes on the quality of life in pediatric tuberculosis survivors and to establish if the changes eventually resolve over a longer period of time. Highlights of the pediatric tuberculosis literature in 2024 are displayed in Table 3.

**Table 3.** Highlight Publications on Tuberculosis in Children in 2024

Title	Impact	Ref.
Bruzadelli Paulino da Costa F, et al. **Mycobacterium tuberculosis infection and tuberculosis disease in the first decade of life: a South African birth cohort study** *Lancet Child Adolesc Health.* 2024	This unique South-African birth cohort provided essential data on TB transmission and disease in a high prevalence setting, and found that the cumulative hazard of tuberculosis disease was as high as 10% at the age of 10 years. The study also documented the efficacy of preventive treatment in children who experienced TST conversion.	[117]
Hesseling A, et al. **Levofloxacin preventive treatment in children exposed to MDR tuberculosis** *N Engl J Med.* 2024	This large pediatric study provided essential data regarding the efficacy of preventive treatment with levofloxacin in children and adolescents with MDR-TB contact.	[42]
Meiwes L, et al. **Whispers in the wind: Face mask sampling for Mycobacterium tuberculosis detection in children with pulmonary tuberculosis.** *J Infect Dis.* 2024	The authors developed a novel method to detect *M. tuberculosis*-specific DNA from face masks in children, with a very low limit of detection (<4 CFU per face mask). Although an exploratory study in 10 children with tuberculosis failed to identify DNA in their face masks, further adaptation of this painless, easy-to-use collection method may be able to increase the diagnostic yield.	[65]
Olbrich L, et al. **Diagnostic accuracy of a three-gene Mycobacterium tuberculosis host response cartridge using fingerstick blood for childhood tuberculosis: a multicentre prospective study in low-income and middle-income countries** *Lancet Infect Dis.* 2024	This large study conducted in geographically-diverse locations showed that analysis of gene signatures in blood can potentially be used to diagnose tuberculosis in children, although the assay's performance was suboptimal.	[120]
Rodriguez-Molino P, et al. **Tuberculosis disease in immunocompromised children and adolescents: a Paediatric Tuberculosis Network European Trials Group multicenter case-control study** *Clin Infect Dis.* 2024	In this large Europe-based cohort, children with immunocompromise were found to have increased rates of non-respiratory TB, severe disease, and long-term sequelae. The study also confirmed that immune-based TB tests show poor performance in this group of patients.	[118]

## NTM-PULMONARY DISEASES

Though opportunistic, infections caused by nontuberculous mycobacteria (NTM) are of growing concern, especially in industrialized countries where the prevalence of NTM disease is often surpassing that of tuberculosis.

A landmark study using 32 years of data from the Danish reference laboratory examining the clinical significance, species distribution, and temporal trends of NTM isolates emphasized the utility of microbiological surveillance as a proxy for monitoring NTM disease in the absence of mandatory notification for NTM in most countries [4]. The results revealed a steady increase in positive NTM cultures, primarily driven by pulmonary isolates. Remarkably, 52% of isolates were associated with clinically significant disease [124, 125]. Annual incidence of possible or definite pulmonary disease increased by 4.6%, largely due to *M. avium* and *M. malmoense*. The latter is a rare but clinically significant cause of NTM lung disease, associated with cavitation and mortality up to 26%, as highlighted in a recent systematic review and meta-analysis of 859 cases [126]. Treatment regimens are mainly based on expert opinion [127], but macrolide- and rifampicin-containing regimens are linked to a higher likelihood of favorable outcomes.

Despite a growing awareness of chronic lung conditions predisposing to NTM lung disease, many patients remain undiagnosed. To guide screening, 12 European NTM experts identified key clinical characteristics through a modified Delphi consensus. They suggest testing for NTM in the presence of 3 or more suggestive symptoms or indicative radiology. Patients with chronic lung disease or those initiating or receiving long-term macrolide therapy might benefit from regular screening [128].

Screening for and diagnosis of NTM lung disease largely relies on often difficult to obtain respiratory tract samples, which contributes to diagnostic delays [129]. Serum anti-glycopeptidolipid (GPL) core IgA antibody testing, an approved diagnostic tool for pulmonary *M. avium* complex (MAC) disease in Japan, proved useful as a test to discriminate NTM disease from colonization in a large bronchiectasis cohort [130, 131].

A promising innovation is a serum-based assay that combines PCR with a clustered regularly interspaced short palindromic repeats (CRISPR)-Cas system to detect circulating MAC cell-free DNA (cfDNA) [132]. This assay yielded diagnostic sensitivity and specificity estimates of over 90% and 97%, respectively. A significant decrease in serial serum MAC cfDNA concentrations was noted at 3 and 6 months after initiation of MAC-directed treatment. While further validation is needed, this tool has the potential to reduce diagnostic delays and enable noninvasive treatment monitoring. These tests, however, do not obviate the need for sputum culture.

There is a growing recognition of the importance of pharmacokinetic-pharmacodynamic (PKPD) data in optimizing NTM therapy: the use of rifampicin in MAC therapy is increasingly questioned, mainly because tolerable rifampicin dosing precludes attaining sufficient PK targets for MAC lung disease [133], while clofazimine might benefit from a loading dose for more effective treatment [134].

Few NTM-specific drugs have been developed, with amikacin liposome inhalation suspension (ALIS) being the exception, licensed for refractory MAC lung disease [135], and under investigation for first-line treatment. Encouraging preliminary results from the ARISE study were presented at the 2024 ATS conference [136]. This study compared the efficacy of ALIS with inhaled empty liposomes, both added to a background regimen of azithromycin and ethambutol. By month 7, culture conversion rates reached 78.8% in the ALIS arm vs 47.1% in the comparator arm, demonstrating the potential of this intervention.

Research into *M. abscessus* remains a critical priority, as this species continues to present a major therapeutic challenge [137].

Progress has been made but many questions remain unanswered, particularly relating to NTM lung disease activity and progression and the need for shorter, better tolerated and effective treatment regimens [138]. A collaborative effort from the NTM community will be required to address these in coming years. Highlights of the NTM disease literature in 2024 are displayed in Table 4.

**Table 4.** Highlight Publications on NTM Diseases in 2024

Title	Impact	Ref.
Darois V, et al. **Toward better cures for *M. abscessus* lung disease.** *Clin Microbiol Rev.* 2024	Elaborate overview discussing preclinical strategies to build all-oral, safe *M. abscessus* treatment regimens as well as advances in clinical development, biomarkers correlating with cure and *M. abscessus* specific clinical trial concepts. An insightful paper for clinicians and researchers alike.	[137]
Loebinger MR, et al. **Patients at risk of nontuberculous mycobacterial pulmonary disease who need testing evaluated using a modified Delphi process by European experts.** *ERJ Open Res.* 2024	Expert consensus providing insights into which clinical characteristics and/or comorbidities should prompt testing for NTM in order to reduce missed diagnoses of NTM lung disease.	[128]
Nguyen MH, et al. **Executive summary: state-of-the-art review: nontuberculous mycobacterial pulmonary disease: patients, principles, and prospects** *Clin Infect Dis.* 2024	Narrative review of foundational principles in the management of NTM lung disease which offers practical guidance – beyond the guidelines - for clinicians caring for these patients covering diagnosis, treatment decisions and prospects.	[138]
van Ingen J, et al. **Rifampicin has no role in treatment of *Mycobacterium avium* complex pulmonary disease and bactericidal sterilising drugs are needed: a viewpoint** *Eur Respir J.* 2024	Critical and insightful viewpoint questioning the role of rifampicin in pulmonary MAC therapy and addressing the need for more PK-PD science in NTM disease.	[133]
Wetzstein N, et al. **Clinical spectrum and relevance of *Mycobacterium malmoense*: Systematic review and meta-analysis of 859 patients.** *J Infect.* 2024	A comprehensive review highlighting the clinical spectrum and significance of disease – pulmonary and extrapulmonary – caused by *M. malmoense.*	[126]

## GAPS AND CHALLENGES

In many ways, there has never been a time for more optimism, but significant challenges still lie ahead to reach the WHO End TB goals [1, 139].

Long-term sequelae of tuberculosis had, for a long time, been barely addressed. Post-tuberculosis mortality is high, especially in PLHIV [140]. In 2023, the proceedings of the 2^nd^ International Post-Tuberculosis Symposium summarized critical knowledge gaps [141, 142]. Two recent scoping reviews address current knowledge about interventions to improve post tuberculosis lung health in adults and children [143, 144]. Host directed therapy (HDT) is one important approach to mitigate pulmonary sequelae of tuberculosis, but there is a paucity of tuberculosis treatment trials and HDT trials addressing post tuberculosis outcomes [145]. N-acetylcysteine (1,200 mg bid for 112 days during tuberculosis treatment) led to no acceleration of culture conversion, but faster recovery of lung function impairment, measured by percentage change in forced vital capacity and forced expiratory volume in the first second [146]. In a trial from India, pulmonary rehabilitation improved parameters assessing dyspnea, quality of life, and mental health indices post-tuberculosis significantly, however improvements in functional capacity were not statically significant [147].

There is a pressing need to speed up diagnosis of tuberculosis and *M. tuberculosis* drug resistance across the spectrum from sub-clinical to clinically detectable tuberculosis to ensure rapid initiation of preventive and therapeutic regimens. A point-of-care test based on easily obtained samples would allow a one-stop “diagnose and treat” approach. There is also a need for biomarkers to determine optimal length of treatment. Innovative trial designs (eg, platform and basket trials) must be harnessed for efficient evaluation of new therapeutics and vaccines. There must then be timely access for patients to these innovations in tuberculosis care.

The tuberculosis community must also address the threat of climate change on tuberculosis control. We will likely see disruption of supply chains, displacement, changing migration patterns, and reduced availability of resources [148]. Failure to prepare now will lead to increasing incidence, higher rates of drug-resistance, loss of the momentum gained in research, and increased global morbidity and mortality.

NTM disease remains poorly understood, and the evidence base to inform diagnosis, treatment, and prevention is scant. Biomarkers are needed to identify individuals who will benefit most from therapy, ie, at highest risk of progression. Randomized clinical trials must be designed and funded as a priority with careful thought given to definition of disease, inclusion criteria, and outcome measures for treatment success as guidelines now rely heavily on observational studies and expert opinion [129].

Finally, in both tuberculosis and NTM disease, it is critical that we work to ensure that the voice of those who have lived with infection is heard. Community engagement has been increasingly recognized as a key element of tuberculosis research, and, to quote the disabilities advocacy movement, it must be “nothing about us without us” [149]. Priorities for tuberculosis and NTM disease research, development, and implementation are shown in Table 5.

**Table 5.** Priorities for Tuberculosis and Non-tuberculous Mycobacteria Disease Research, Development, and Implementation

Area of Action	Strategies and Targets
Prevention	An improved understanding of the immune response to *Mycobacterium tuberculosis* and NTM species A clear understanding of the different types of controls needed to assess diagnostic tests A re-evaluation of non-inferiority trials and the role of standard-of-care treatments that have an unusually low cure rate A need for appropriate controls in estimating post-tuberculosis lung disease Vaccines with high efficacy for protection against mycobacterial diseases Improve nutritional status of patients on TB and NTM-ID treatment and TB household contacts Test to predict more accurately the risk of progression from infection to disease Global access to rifapentine Improved diagnosis of LTBI and treatment coverage with TPT
Diagnosis	Rapid point of care diagnostic test using easily-accessible samples Test to predict *M. tuberculosis* and NTM culture conversion Non-sputum-based markers of treatment response Test to personalize end of treatment AI based radiation-free imaging for treatment monitoring Test to predict treatment adverse events Rapid implementation of resistance testing to novel drugs (culture-free) Improved access to new diagnostic tools in affected communities Improved case finding and treatment coverage
Treatment	Personalized medicine approaches for TB treatment Development of evidence-based regimens for the treatment of bedaquiline-resistant and XDR-TB Clinical trials to improve management of extra-pulmonary TB Improved access to new drugs and regimens for drug-resistant TB
Post disease care	Inclusion of post disease care in programmatic TB and NTM-ID management and advocacy Inclusion of post-disease endpoints in clinical trials Development of strategies and interventions to prevent post mycobacterial lung disease
Pediatric TB care	Improvement of the yield of current non-sputum-based diagnostics to match that of respiratory samples Development of new diagnostic tools for pediatric TB with greater sensitivity than existing tests Inclusion of children and adolescents in trials of new anti-TB drugs Further development of child-friendly medications and better availability thereof
Policy and funding	Sustained funding mechanisms for TB programs, care and research Prioritizing co-production in research and advocacy in policy work
NTM	Gather evidence on role of preventive measures relating to environmental exposure avoidance Markers to discriminate colonization from infection and disease Determine break-points for new/repurposed NTM drugs Fund research into new/repurposed drug pipeline Shorter, effective, better tolerated drug regimens

## CONCLUSIONS

2024 was a year with manifold scientific progress in the areas of tuberculosis and NTM disease. Selected highlights of the literature on adult tuberculosis are shown in Table 6 and key changes in WHO recommendations for the management of tuberculosis in Table 7.

**Table 6.** Highlight Publications on Adult Tuberculosis in 2024

Title	Impact	Ref.
Cevik M, et al. **Bedaquiline-pretomanid-moxifloxacin-pyrazinamide for drug-sensitive and drug-resistant pulmonary tuberculosis treatment: a phase 2c, open-label, multicentre, partially randomised controlled trial.** *Lancet Infect Dis.* 2024	A randomized phase IIc trial with 3 arms: 1 (control). Isoniazid and rifampicin for 6 months with pyrazinamide and ethambutol (HRZE) in the first 2 months in patients with drug-susceptible tuberculosis; 2. Bedaquiline, pretomanid, moxifloxacin and pyrazinamide (BPaMZ)for 4 months in patients with drug-susceptible tuberculosis and 3. BPaMZ for 4 months in patients with drug-susceptible tuberculosis. Despite bacteriological efficacy, participants receiving 4 months of BPaMZ withdrew more often from the trial due to adverse hepatic events than did those in the HRZE arm. Results from this trial will likely not lead to recommendations for treatment shortening in drug-susceptible tuberculosis with the BPaMZ regimen or for a recommendation of BPaMZ for 6 m treatment in drug-resistant tuberculosis due to an inferior safety profile of the BPaMZ regimen compared to other alternatives.	[89]
Chang VK, et al. **Risk-stratified treatment for drug-susceptible pulmonary tuberculosis**. *Nat Commun.* 2024	Based on data from the ACTG 301 trial the authors developed a simple risk score based on the cycle threshold in Xpert-Ultra detecting *M. tuberculosis* DNA in sputum, the area on a chest x-ray showing infiltrates and the blood levels of rifapentine to propose 3 different strata: easier-, moderately-harder, or harder-to-treat tuberculosis. The results suggest that the easier-to-treat subgroup may be eligible for treatment shortening while the harder-to-treat subgroup may need higher doses or longer treatment. The proposed risk score needs to be validated independently.	[93]
Coussens AK, et al. **Classification of early tuberculosis states to guide research for improved care and prevention: an international Delphi consensus exercise.** *Lancet Respir Med.* 2024	This is a consensus-derived proposal for a new framework of classification for tuberculosis that accommodates key disease states but is sufficiently simple to support pragmatic research and implementation. The proposal distinguishes disease from infection by the presence of macroscopic pathology and defines two asymptomatic and two clinical tuberculosis states on the basis of reported symptoms or signs of tuberculosis, further differentiated by likely infectiousness.	[12]
Diacon AH, et al. **A first-in-class leucyl-tRNA synthetase inhibitor, ganfeborole, for rifampicin-susceptible tuberculosis: a phase 2a open-label, randomized trial.** *Nat Med*. 2024	Successful early bactericidal activity study with a novel compound from a new class of anti-tuberculosis medicines providing justification for this compound to move forward to Phase IIb of clinical development. The study provides novel information for changes in metabolic pathways during the early phase of therapy documented by alterations in the mRNA expression profiles corresponding to decreasing inflammation shown on [1^8^F]FDG-PET/CT.	[94]
Duong T, et al. **A Meta-Analysis of Levofloxacin for Contacts of Multidrug-Resistant Tuberculosis.** *NEJM Evid.* 2025	A metanalysis of the two trials using levofloxacin (V-QUIN and TB-CHAMP) documents close to 60% risk reduction using levofloxacin (for 6 months) in adults and children contacts to RR-TB to develop active TB after 54 weeks.	[43]
Gausi K, et al. **High-Dose Isoniazid Lacks EARLY Bactericidal Activity against Isoniazid-resistant Tuberculosis Mediated by katG Mutations: A Randomized Phase II Clinical Trial.** *Am J Respir Crit Care Med.* 2024	This EBA trial investigated the role of high dose isoniazid (15 – 20 mg/kg) in strains containing a katG mutation (>95% of all isoniazid-resistant strains in Europe). Almost no EBA was documented in the given dose range, strongly calling the use of high dose isoniazid in standardized regimens into question.	[113]
Korotych O, et al. **Effectiveness and safety of modified fully oral 9-month treatment regimens for rifampicin-resistant tuberculosis: a prospective cohort study.** *Lancet Infect Dis.* 2024	Results from this, multi-country, prospective, open label study under leadership of the WHO Europe region office, with more than 2500 participants receiving a 9m all-oral treatment regimen consisting of bedaquiline, levofloxacin, linezolid, clofazimine and cycloserin show 83% treatment success in patients with MDR/RR-TB under operational research conditions. Successful treatment outcomes in two small additional study arms with bedaquiline, linezolid, levofloxacin, delamanid and pyrazinamide (n=73) and bedaquiline, linezolid, levofloxacin, delamanid and clofazimine (n=23) showed 89% and 100% successful treatment outcomes.	[105]
Lienhardt C, et al. **Target regimen profiles for tuberculosis treatment.** *Bull World Health Organ.* 2024	A group of international experts developed target regimen profiles (TRP) for the treatment any type of tuberculosis. These TRPs will provide useful guidance for tuberculosis treatment developers to produce regimens that are quality-assured, affordable and widely available, and that meet the needs of affected populations	[95]
Nyang'wa BT, et al. **Short oral regimens for pulmonary rifampicin-resistant tuberculosis (TB-PRACTECAL): an open-label, randomised, controlled, phase 2B-3, multi-arm, multicentre, non-inferiority trial.** *Lancet Respir Med.* 2024	Final report of the Phase IIc/III TB-PRACTECAL trials results from MSF showing superiority of the 6-month BPaLM treatment regimen over standard of care in MDR/RR-TB. The results of this trial have been part of the basis for changes of the WHO recommendations for the management of DR-TB in 2022.	[101]
Stuck L, et al. **Prevalence of subclinical pulmonary tuberculosis in adults in community settings: an individual participant data meta-analysis.** *Lancet Infect Dis.* 2024	This individual patient data meta-analysis concludes that in high-incidence settings, asymptomatic tuberculosis could contribute considerably to the tuberculosis burden and to *Mycobacterium tuberculosis* transmission. The majority of people in the community who have pulmonary tuberculosis do not report cough and a quarter of those not reporting any cough have positive sputum smears, suggesting infectiousness. A quarter of persons affected by pulmonary tuberculosis report no tuberculosis-suggestive symptoms at all.	[14]

**Table 7.** Key Recommendations for Tuberculosis Prevention, Diagnosis, and Treatment by the World Health Organization (WHO) Issued in 2024

Recommendations	Ref.
***Mycobacterium tuberculosis* antigen-based skin tests (TBST)** may be used to test for tuberculosis infection.	[150]
The following alternative **tuberculosis preventive treatment** options may be used regardless of HIV status: a **1-month regimen of daily rifapentine plus isoniazid or 4 months of daily rifampicin**.	[150]
In **adults in the general population** who had either signs or symptoms of tuberculosis or chest radiograph with lung abnormalities or both, the **Xpert MTB/RIF or Xpert Ultra may replace culture** as the initial test for pulmonary tuberculosis.	[151]
Among individuals **aged 15 years and older** in populations in which tuberculosis screening is recommended, **computer-aided detection software programs may be used in place of human readers for interpreting digital chest X-rays for screening** and triage for tuberculosis disease.	[21]
In **adults and children** with signs and symptoms of **extrapulmonary TB, Xpert MTB/RIF may be used in lymph node aspirate, lymph node biopsy, pleural fluid, peritoneal fluid, pericardial fluid, synovial fluid, or urine specimens as the initial diagnostic test** rather than smear microscopy/culture. In adults and children with signs and symptoms of TB meningitis, Xpert MTB/RIF or Xpert Ultra should be used in cerebrospinal fluid (CSF) as an initial diagnostic test for TB meningitis rather than smear microscopy/culture.	[151]
**People living with HIV should** be systematically **screened for tuberculosis disease at each visit** to a health facility. Among **adults and adolescents living with HIV**, systematic **screening for tuberculosis** disease should be conducted using the WHO-recommended **four symptom screen,** and those who report any one of the symptoms of **current cough, fever, weight loss, or night sweats** may have tuberculosis and should be evaluated for tuberculosis and other diseases.	[21]
In **outpatient settings:** WHO suggests using **LF-LAM** to assist in the diagnosis of active tuberculosis in **HIV-positive adults, adolescents, and children** with signs and symptoms of TB *(pulmonary and/or extrapulmonary) or seriously ill; and* irrespective of signs and symptoms of TB and with a CD4 cell count of less than 100 cells/mm^3^	[21]
In **adults and adolescents with HIV** who have signs or symptoms or screened positive for tuberculosis, or seriously ill, or have advanced HIV disease, **concurrent testing using low-complexity automated NAATs on respiratory samples and LF-LAM on urine** should be used as the initial diagnostic strategy for diagnosing TB rather than low-complexity automated NAATs on respiratory samples alone. In **children** who have signs or symptoms or screened positive for pulmonary tuberculosis, **concurrent testing using low-complexity automated NAATs on respiratory samples and stool** should be used as the initial diagnostic strategy for diagnosing tuberculosis rather than low-complexity automated NAATs on respiratory or stool samples alone.	[152]
The **6-month BPaLM** regimen, comprising bedaquiline, pretomanid, linezolid (600 mg) and moxifloxacin, may be used programmatically in place of 9-month or longer (>18 months) regimens, in patients (aged ≥14 years) with MDR/RR-tuberculosis who have not had previous exposure to bedaquiline, pretomanid, and linezolid (defined as >1 month exposure). This regimen may be used without moxifloxacin (BPaL) in the case of documented resistance to fluoroquinolones (in patients with pre-XDR-tuberculosis). The **6-month BDLLfxC regimen**, composed of bedaquiline, delamanid, linezolid (600 mg), levofloxacin, and clofazimine, may be used programmatically in place of 9-month or longer (>18 months) regimens, in all patients with MDR/RR-tuberculosis who have not had previous exposure to bedaquiline, delamanid, and linezolid (defined as >1 month exposure). The regimen may be used without either levofloxacin or clofazimine depending on fluoroquinolone DST results - BDLLfxC can be initiated without delay in case of unknown FQ-resistance at time of diagnosis of RR-tuberculosis (and may be continued with both levofloxacin and clofazimine if FQ-DST results cannot be obtained); BDLLfx is continued for FQ-sensitive TB; BDLC for FQ-resistant TB. The available evidence included children, adolescents, pregnant and breastfeeding women, flagging the possible use of the regimen in these population groups. The use of the **modified 9-month, all-oral regimens (BLMZ, BLLfxCZ and BDLLfxZ)** is preferred over currently recommended longer (18-month) regimens in patients with MDR/RR-TB who have not had previous exposure to bedaquiline, delamanid, and linezolid (defined as >1-month exposure) and in whom resistance to fluoroquinolones has been excluded. Among these regimens, using BLMZ is suggested over BLLfxCZ, and BLLfxCZ is suggested over BDLLfxZ. Access to rapid DST for ruling out fluoroquinolone resistance is required before beginning one of these regimens.	[109]
**Co-administration of both MDR-tuberculosis and HCV treatments is favored** for better outcomes and cost-effectiveness over delaying HCV treatment until after MDR/RR-TB treatment completion. This applies to all patients with confirmed MDR/RR-tuberculosis and HCV.	[153]

Tuberculosis has remained an important global health problem, with little change in the estimated case numbers since being declared a global emergency in 1994. *Mycobacterium tuberculosis* drug-resistance is increasingly recognized as a global threat [10]. Co-morbidities remain an important problem. While HIV co-infection has fallen dramatically world-wide with the use of antiretroviral therapy, undernutrition, alcohol use disorders, smoking, and diabetes mellitus remain common and important risk factors for tuberculosis [1]. Our limited understanding of the immunology of tuberculosis remains a problem, with many misconceptions and an over-reliance on measurement of interferon-γ as a proxy for tuberculosis infection. The year 2024 has seen greater application of DNA-based tests, especially in children, using samples like stool that are more readily accessible than sputum [73].

2024 was the year when new treatment regimens for DR-TB went mainstream. BPaL(M) has become the standard of care in MDR/RR-TB and pre-XDR-TB [154–156] in many countries, although the availability of pretomanid is still limited [104]. However, the growing proportions of bedaquiline-resistant *M. tuberculosis* strains are alarming [5, 157, 158]. The 6-month BEAT-TB regimen (BLfxL with D or Cfz) is now also advocated by the WHO – although the study results are not yet published in 2024 and there are no trials comparing this regimen with BPaL(M) [109].

By the end of 2024 there was still a substantial gap in the availability of either molecular prediction of drug resistance by tNGS or WGS and phenotypic DST to determine if bedaquiline, pretomanid, or delamanid are effective [159]. Also, the high frequency of toxicity attributed to linezolid continues to be a problem for all shorter treatment regimens against DR-TB [160, 161].

Six months of levofloxacin as preventive therapy after exposure to MDR/RR-TB has gained traction by the amalgamation of 2 studies [41–43]. As fluoroquinolones are often used for respiratory infections, acquired resistance may jeopardize the new regimens in the longer term.

While enormous progress has been made in mycobacterial disease research in 2024, not enough resources are being allocated to this global pandemic. Without further concerted efforts, we will not achieve the goals of the aspirational End TB targets for reducing morbidity and mortality related to tuberculosis. Although less of a public health emergency, NTM lung disease is becoming increasingly recognized and could potentially become a substantial burden to the healthcare system. There remains an important knowledge gap in proper diagnosis and management of this condition, which urgently requires addressing through more research investment.
